# TTCC-2019-02: real-world evidence of first-line cetuximab plus paclitaxel in recurrent or metastatic squamous cell carcinoma of the head and neck

**DOI:** 10.3389/fonc.2023.1226939

**Published:** 2023-08-01

**Authors:** Jordi Rubió-Casadevall, Beatriz Cirauqui Cirauqui, Javier Martinez Trufero, Maria Plana Serrahima, Almudena García Castaño, Alberto Carral Maseda, Lara Iglesias Docampo, Pedro Pérez Segura, Isaac Ceballos Lenza, Vanesa Gutiérrez Calderón, José Fuster Salvà, Carolina Pena Álvarez, Irene Hernandez, Edel del Barco Morillo, Manuel Chaves Conde, Joaquina Martínez Galán, Marisa Durán Sánchez, Vanesa Quiroga, Eugenia Ortega, Ricard Mesia

**Affiliations:** ^1^ Medical Oncology Department, Institut Català d’Oncologia Girona, Girona Biomedical Research Institute (IDIBGI), Girona, Spain; ^2^ Medical Oncology Department, Institut Català d’Oncologia Badalona, B-ARGO Group, IGTP, Badalona, Spain; ^3^ Medical Oncology Department, Hospital Universitario Miguel Servet, Zaragoza, Spain; ^4^ Medical Oncology Department, Institut Català d’Oncologia (ICO-Hospitalet), IDIBELL, Hospitalet de Llobregat, Llobregat, Spain; ^5^ Medical Oncology Department, Hospital Universitario Marqués de Valdecilla, Santander, Spain; ^6^ Medical Oncology Department, Hospital Universitario Lucus Augusti, Lugo, Spain; ^7^ Medical Oncology Department, Hospital Universitario 12 de Octubre, Madrid, Spain; ^8^ Medical Oncology Department, Hospital Clínico San Carlos, IdISSC, Madrid, Spain; ^9^ Medical Oncology Department, Hospital Universitario de Canarias, San Cristóbal de La Laguna, Spain; ^10^ UGCI Oncol. Hospital Universitario Regional y Virgen Victoria, IBIMA, Málaga, Spain; ^11^ Medical Oncology Department, Hospital Universitario Son Espases, Palma de Mallorca, Spain; ^12^ Medical Oncology Department, Centro Oncológico Galicia, A Coruña, Spain; ^13^ Medical Oncology Department, Hospital Universitario de Navarra, Instituto de Investigación Sanitaria de Navarra (IdiSNA), Pamplona, Spain; ^14^ Medical Oncology Department, Hospital Universitario de Salamanca, IBSAL, Salamanca, Spain; ^15^ Medical Oncology Department, Hospital Universitario Virgen de Valme, Sevilla, Spain; ^16^ Medical Oncology Department, Hospital Universitario Virgen Nieves, Instituto de Investigación Biosanitaria ibs, Granada, Spain; ^17^ Spanish Group of Head and Neck Cancer Treatment (TTCC), Madrid, Spain

**Keywords:** squamous cell carcinoma of head and neck, cetuximab, paclitaxel, platinum ineligible, frail patients

## Abstract

**Objectives:**

The aim of this study was to confirm the efficacy of the ERBITAX scheme (paclitaxel 80 mg/m^2^ weekly and cetuximab 400 mg/m^2^ loading dose, and then 250 mg/m^2^ weekly) as first-line treatment for patients with recurrent/metastatic squamous cell carcinoma of the head and neck (SCCHN) who are medically unfit for cisplatin-based (PT) chemotherapy.

**Materials and methods:**

This retrospective, non-interventional study involved 16 centers in Spain. Inclusion criteria were to have started receiving ERBITAX regimen from January 2012 to December 2018; histologically confirmed SCCHN including oral cavity, oropharynx, hypopharynx, and larynx; age ≥18 years; and platinum (PT) chemotherapy ineligibility due to performance status, comorbidities, high accumulated dose of PT, or PT refractoriness.

**Results:**

A total of 531 patients from 16 hospitals in Spain were enrolled. The median age was 66 years, 82.7% were male, and 83.5% were current/former smokers. Patients were ineligible to receive PT due to ECOG 2 (50.3%), comorbidities (32%), PT cumulative dose ≥ 225 mg/m^2^ (10.5%), or PT refractoriness (7.2%). Response rate was 37.7%. Median duration of response was 5.6 months (95% CI: 4.4–6.6). With a median follow-up of 8.7 months (95% CI: 7.7–10.2), median PFS and OS were 4.5 months (95% CI: 3.9–5.0) and 8.9 months (95% CI: 7.8–10.3), respectively. Patients treated with immunotherapy after ERBITAX had better OS with a median of 29.8 months compared to 13.8 months for those who received other treatments. The most common grade ≥ 3 toxicities were acne-like rash in 36 patients (6.8%) and oral mucositis in 8 patients (1.5%). Five (0.9%) patients experienced grade ≥ 3 febrile neutropenia.

**Conclusion:**

This study confirms the real-world efficacy and tolerability of ERBITAX as first-line treatment in recurrent/metastatic SCCHN when PT is not feasible. Immunotherapy after treatment with ERBITAX showed remarkable promising survival, despite potential selection bias.

## 1 Introduction

Squamous cell carcinoma of the head and neck (SCCHN) is one of the most frequently diagnosed cancers worldwide. More than half of patients diagnosed with locally advanced disease will recur within 5 years (1, 2). Most patients receive platinum-based chemotherapy as first-line treatment for recurrent/metastatic disease, which has a modest impact on survival (3, 4). Recently, the use of immune-checkpoint inhibitors (ICIs) such as nivolumab (5) and pembrolizumab (6) has shown to be effective in the treatment of advanced patients refractory to platinum-based chemotherapy. Likewise, the use of these drugs in the first line can also provide a substantial benefit according to programmed death ligand 1 (PD-L1) combined positive score (CPS) (7).

Recurrent or metastatic SCCHN is challenging because most of the patients have poor performance status, which is accompanied by nutritional disorders, abuse of alcohol or tobacco, or comorbidities (8, 9). Additionally, some patients may have a high accumulated dose of platinum from previous treatments for localized disease or may be considered refractory to platinum. Therefore, many patients are not eligible for platinum-based chemotherapy because they are frail enough to withstand the treatment approach or are expected to not respond to platinum. Although most patients unfit for platinum may have CPS >1, and now can start with a pembrolizumab-based first-line therapy (7), many patients can be CPS < 1 and will not be eligible for this therapeutic approach.

A regimen with the combination of cetuximab (CX) and weekly paclitaxel (PX), named ERBITAX, has shown activity and is well tolerated in patients with SCCHN with poor prognosis, including those for whom platinum is contraindicated (10). Therefore, this is a common option for treating medically unfit or platinum-resistant patients with SCCHN.

The study aimed to describe the real-world characteristics, treatment compliance, safety, and survival outcomes of patients with SCCHN who were unfit to receive platinum and received ERBITAX as first-line treatment for recurrent or metastatic disease in Spain.

## 2 Patients and methods

### 2.1 Study design

The TTCC-2019-02 was an observational, retrospective study conducted in 16 hospitals in Spain (**Supplementary Data**).

This study used secondary data retrieved from medical records. The medical records included all clinical variables required to perform the analysis, and accessing additional sources was not necessary. The assignment of a patient to a specific therapeutic strategy had already been decided in advance by the routine clinical practice of medicine, and the decision to prescribe the ERBITAX scheme was clearly dissociated from the decision to include a patient in the study. No additional interventions different from the standard clinical practice were applied to the patients, either for diagnosis or due to follow-up reasons. Epidemiological methods were used to analyze the data.

The study was conducted according to local regulations, ICH - Good Clinical Practice (R2), and the International Conference on Harmonisation and Declaration of Helsinki. The protocol was approved by the ethics committee of the Hospital Universitari de Bellvitge on 23 October 2020 (IEC code: EPA019/20). The protocol, amendments, and informed consent forms were approved by the institutional review board or ethics committee of each study site before study initiation. All patients were asked to provide written informed consent to participate, although an informed consent exemption was considered in cases when the effort to obtain informed consent was beyond reasonable and feasible (i.e., dead patients).

### 2.2 Study population

Patients aged ≥18 years with histologically confirmed recurrent or metastatic SCCHN, including the oral cavity, oropharynx, hypopharynx, and larynx; considered unfit for standard platinum-based chemotherapy; and who received at least one dose of weekly paclitaxel (starting dose of 80 mg/m^2^) and cetuximab (loading dose of 400 mg/m^2^, followed by 250 mg/m^2^) as first-line treatment in the recurrent or metastatic setting were eligible. Paclitaxel was administered weekly (i.e., days 1, 8, 15, 21…). Cetuximab could have been switched to biweekly during the maintenance phase. The included patients had to have started the ERBITAX regimen between January 2012 and December 2018. Unfit criteria for platinum included the following: poor Eastern Cooperative Oncology Group performance status (ECOG PS), comorbidities, and high accumulated dose of platinum or platinum refractoriness, defined as early disease progression (less than 6 months) to previous platinum-based chemotherapy in locally advanced setting (11, 12). Patients with unknown primary tumor, nasopharyngeal cancer, and non-squamous head and neck tumor, or ECOG >2 were excluded from the study.

Once the patients who had received at least one complete cycle of ERBITAX were identified, all cases from each center were included to avoid selection bias.

### 2.3 Objectives

The primary objective was to estimate the progression-free survival (PFS) in patients treated with ERBITAX as the first-line treatment for recurrent and/or metastatic SCCHN.

The secondary objectives were to determine the efficacy of ERBITAX measured by the best overall response (BOR), objective response rate (ORR), disease control rate (DCR), duration of response (DoR), and overall survival (OS); to establish potential prognostic factors associated with survival; and to characterize the safety profile by means of treatment compliance and toxicities.

### 2.4 Endpoints

Descriptive baseline characteristics to define the study population included demographic (i.e., age, sex, and ECOG PS), habits (i.e., smoking and enolic habit), HPV status (through p16 IHQ and DNA-HPV analysis), and pathological (i.e., stage and location) endpoints.

The primary endpoint, PFS, was defined as the time from the start of the study treatment to the date of progression or death, whichever occurred first. Patients without a PFS event were censored on the date of the last radiological evaluation or on the date of the last study treatment if the tumor response was not evaluated later. If no PFS event was observed prior to the start of second-line treatment, the patient was censored at the date of second-line treatment.

Secondary endpoints for efficacy included the following: BOR, classified as complete response (CR), partial response (PR), standard disease (SD), or progressive disease (PD) according to RECIST 1.1; ORR, defined as the proportion of patients with CR or PR; DCR, defined as the proportion of patients with CR, PR or SD; DoR, defined as the time from the first occurrence of response until PD or death, whichever occurs first; and OS, defined as time from the start of the study treatment until the date of death due to any cause. For alive patients, the OS time was censored at the last date known to be alive. Long-term survivors for stratified analysis of OS were considered those who were alive and disease-free 2 years after the start of the study treatment. Stratified analysis of subgroups according to baseline characteristics was used to determine the potential prognostic factors for efficacy.

Treatment compliance was measured using relative dose intensity (RDI), defined as the amount of drug administered per unit of time expressed as the fraction of that established in the standard regimen, rate of dose interruptions, modifications, and discontinuations. RDI was calculated as follows: RDI = 100*(DTD/STD) where DTD (Delivered Total Dose) is the total amount of drug actually administered over chemotherapy course in mg/m^2^ and STD (Standard Total Dose) is the total standard amount of drug for administration over chemotherapy course in mg/m^2^.

Secondary endpoints for safety included the rate of adverse events (AEs) that fulfilled any of the following criteria: AEs that were grade ≥3, led to discontinuation or dosage reduction of paclitaxel and/or cetuximab, or met criteria for serious AE (SAE). AEs were classified and graded according to the National Cancer Institute Common Toxicity Criteria for Adverse Events (NCI CTCAE) version 5.0.

The frequency of assessments followed the standard clinical practice. Preventive measures to identify patient duplicates were implemented, including cross-checking of variables such as birth date, sex, center, or diagnosis. We consider death as a cause of treatment interruption when the progression has not been objectified or clearly reflected in the clinical reports, although it is possible it would have occurred, and it is understood that the treatment does not have to be the cause of death.

### 2.5 Statistical analysis

Descriptive statistics and frequency tables were provided for all baseline, efficacy, and safety variables as appropriate. Continuous variables were summarized using mean, standard deviation, range, and median values. Frequency counts and percentages of participants within each category were provided for categorical data. The response percentages were estimated by binomial proportion using 95% confidence intervals (CIs) or full-range intervals. The time-to-event endpoints were estimated using the Kaplan–Meier method and log-rank test was used to obtain the median, CIs. Cox regression analysis was used to statistically compare between patient subgroups. Patients without documented progression or death at the time of analysis were censored on the last date of tumor evaluation. Logistic regression and Cox regression models were used to analyze the association between baseline characteristics, the treatment and disease outcomes. Multivariate models were used to estimate the hazard ratios (HR) for OS. All statistical analyses were performed using R (version 3.6.3 [2020-02-29] “Holding the Windsock,” The R Foundation for Statistical Computing, Vienna, Austria). Figures and tables were generated using RStudio (Version 1.2.5033 2009-2019 RStudio, Inc., Boston, MA, USA). All statistical tests were two-tailed with an alpha of 0.05.

The sample size calculation was based on the estimated number of patients with recurrent and/or metastatic SCCHN who were treated with ERBITAX in the first-line setting in Spain. The study planned to enroll at least 500 patients, which was considered a representative sample for the population of patients with SCCHN treated with ERBITAX. Assuming a total of 500 evaluable patients for PFS, and a censoring rate of 20%, the width (precision) of the 95% CI is 0.032 when the estimated hazard rate was 0.165 (95% CI: 0.1492–0.1816). The corresponding 95% CI for an estimated median PFS of 4.2 months is 3.8–4.6 months, which provides a considerably more precise estimate of the median PFS than previous reports (10).

## 3 Results

### 3.1 Patient disposition

**Figure 1:**
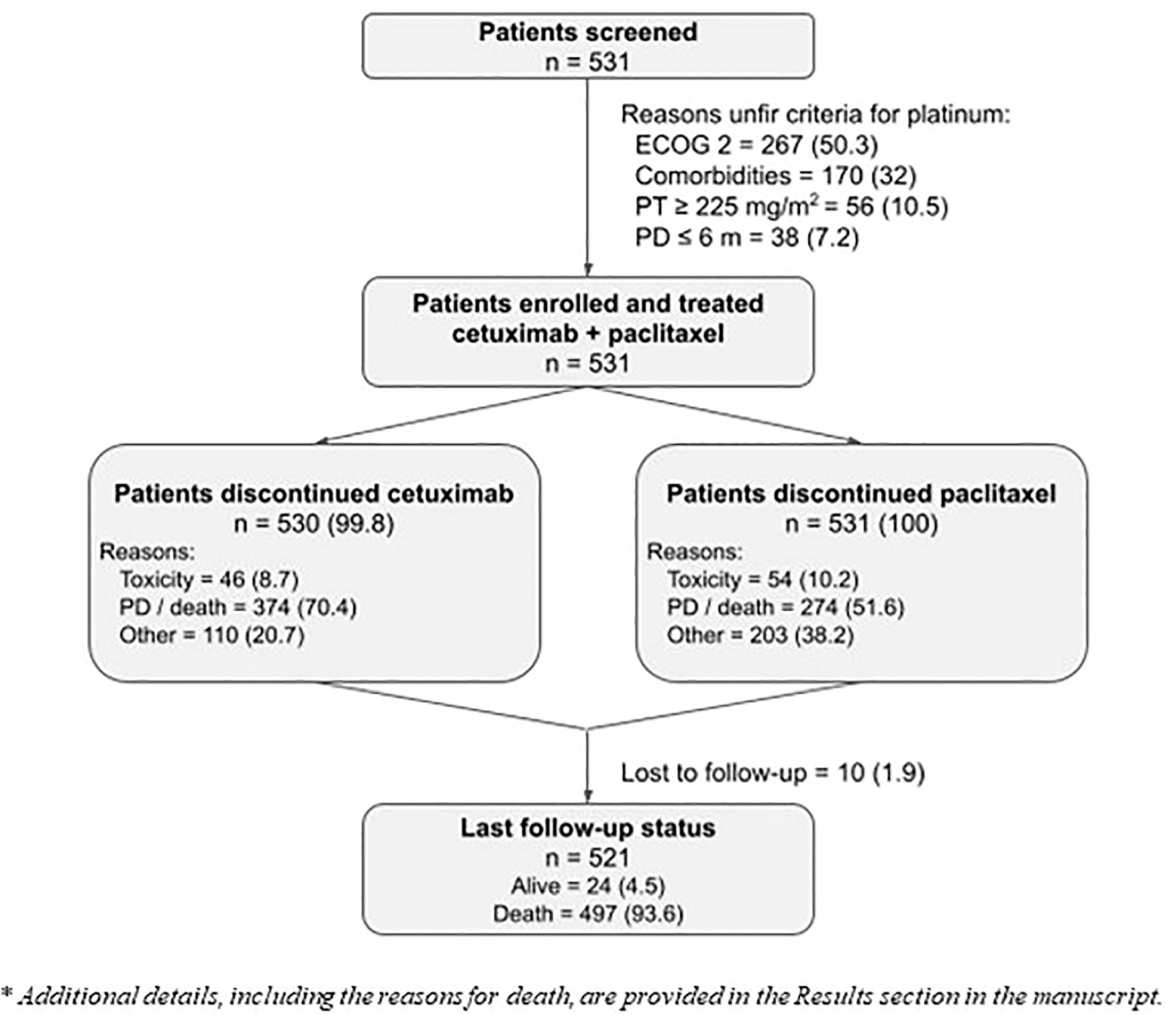
Patient disposition. Numbers are expressed as number of patients and percentage; n (%). The reasons for unfit criteria were ranked with ECOG 2 being the first evaluated, comorbidities second, and platinum ineligibility due to high accumulated dose or refractoriness as third.

A total of 531 patients were enrolled in this study between December 2020 and September 2021 (**Figure 1**). The number exceeded the minimum planned sample size by approximately 5% to allow inclusion of all patients treated in each center, thus avoiding biasing. All patients who met the eligibility criteria and received at least one dose of ERBITAX were evaluable for the study.

### 3.2 Demographic and baseline clinical characteristics

The median age of our sample was 66 years (range: 35–92). Most patients were male (82.7%), were smokers or former smokers (83.5%), had an ECOG score of 2 (50.3%), and had stage IVa-b disease at diagnosis (58.9%). The most common primary tumor sites were oral cavity (36.2%), larynx (30.9%), oropharynx (19.2%) and hypopharynx (13.7%). Previous systemic chemotherapy before recurrent/metastatic setting was administered to 333 (62.7%) patients (**Table 1**). Previous taxanes during induction were administered to 139 (26.2%) patients. Regarding the ineligibility criteria for PT, the distribution of patients was as follows: ECOG 2, 267 patients (50.3%); comorbidities, 170 patients (32%); PT cumulative dose ≥ 225 mg/m^2^, 56 patients (10.5%); PT refractoriness, 38 patients (7.2%).

**Table 1:** Baseline characteristics.

Characteristics; unit		TTCC-2019-02 *N* = 531
Median age (range); years		66 (35–92)
Sex, *n* (%)	Male	439 (82.7)
Tumor location, *n* (%)	Oral cavity	192 (36.2)
	Oropharynx	102 (19.2)
	Larynx	164 (30.9)
	Hypopharynx	73 (13.7)
ECOG PS; *n* (%)	0	18 (3.4)
	1	246 (46.3)
	2	267 (50.3)
Stage at diagnosis AJCC 7th edition; *n* (%)	I	25 (4.7)
	II	39 (7.3)
	III	89 (16.8)
	IV a-b	313 (58.9)
	IV c	55 (10.4)
	UK	10 (1.9)
Smoker or tobacco use; *n* (%)	Never smoker	60 (11.3)
	Former	226 (42.6)
	Current smoker	217 (40.9)
	UK	28 (5.3)
Enolic habit; *n* (%)	Never drink	122 (23)
	Former	121 (22.8)
	Current drinker	210 (39.5)
	UK	78 (14.7)
Previous treatments; *n* (%)	No previous treatment	57 (10.7)
	Chemotherapy based	5 (1.1)
	Radiotherapy based	173 (36.5)
	Surgery based	296 (62.4)
PD-L1 status; *n* (%)	Positive	26 (4.9)
	Negative	95 (17.9)
	UK	2 (0.4)
	Not performed*	408 (76.8)
Median time from diagnosis to ERBITAX (range); months		13 (0–281)

*Patients were treated between 2012 and 2018. Many patients had no PD-L1 determined as treatment with immunotherapy was not an option by that time and this was not standard care practice.

ECOG PS, Eastern Cooperative Oncology Group performance status; HPV, human papillomavirus; PD, progression; PD-L1, programmed death ligand 1; PT, platinum; UK, unknown.

### 3.3 Treatment compliance

**Figure 2:**
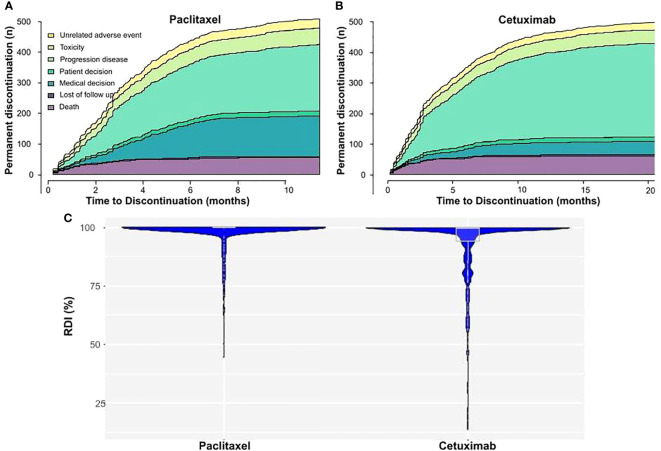
Cetuximab and paclitaxel treatment compliance. **(A)** Cetuximab and **(B)** Paclitaxel permanent discontinuations through time. The number of discontinuations is coloured according to the cause of discontinuation. **(C)** Relative dose intensity (RDI) for cetuximab and paclitaxel. The width of the plot in the X axis represents the number of patients with those RDI levels.

At the data cutoff, most patients discontinued the study treatment, only 1 patient continued treatment with cetuximab, and none with paclitaxel (**Figure 1**). The most common cause of permanent discontinuation was disease progression (59% for CX and 41.1% for PX; percentages vary between treatments due to patients who progressed during cetuximab maintenance monotherapy). Cetuximab and paclitaxel were discontinued due to toxicity in 8.7% and 10.2% of patients, respectively (**Figures 2A, B**). Some patients discontinued one or both drugs due to medical decisions, generally after a long period of treatment, to avoid future toxicities, such as neurotoxicity in the case of PX. Some patients refused to continue with the treatment with absence of adverse events or other documented reasons that would have led to treatment discontinuation. The median duration of treatment with cetuximab was 3.5 months (95% CI: 3.0–4.1) and that with paclitaxel was 2.8 months (95% CI: 2.7–3.2). Dose reductions and temporary interruptions of cetuximab (6.6% and 13.9%) and paclitaxel (7.7% and 13.6%) were required to manage treatment-emergent toxicities. Overall, 219 (41.4%) patients did not achieve 100% RDI for cetuximab, and 66 (12.4%) patients did not achieve 100% RDI for paclitaxel (**Figure 2C**).

### 3.4 Efficacy

The locally assessed ORR was 37.7% (95% CI: 33.5–41.9); 37 (7%) patients achieved CR, 163 (30.7%) achieved PR, 90 (17%) achieved stable disease, 118 (22.2%) progressed, and 123 (23%) patients were not radiologically evaluated. DCR was 54.6% (95% CI: 50.3–58.9). The median DoR for the 200 patients who experienced a response was 5.6 months (95% CI: 4.8–6.6). The median duration of SD was 3.2 months (95% CI: 2.4–3.8).

**Figure 3:**
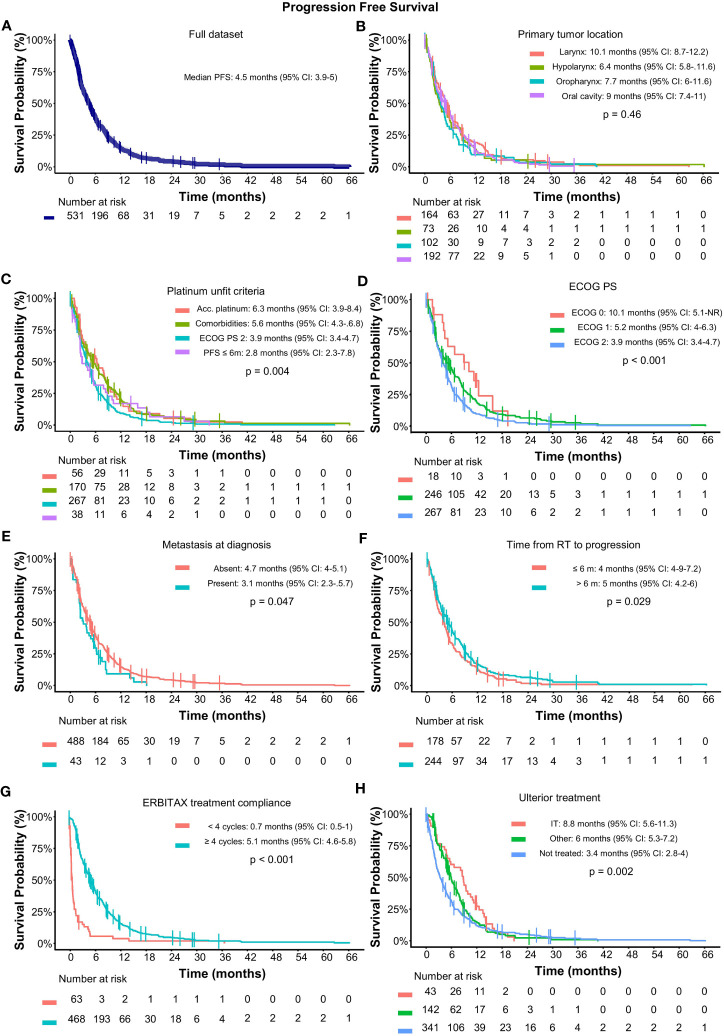
Progression-free survival in SCCHN treated with cetuximab and paclitaxel as first-line treatment. **(A)** PFS for the full dataset; Subgroup analysis of PFS according to **(B)** primary tumor location; **(C)** unfit criteria; **(D)** ECOG PS; **(E)** metastatic status at diagnosis; **(F)** time from radiotherapy; **(G)** ERBITAX treatment compliance; and **(H)** ulterior treatment. Median PFS and significance by cox model are displayed within the graphics. Acc, accumulated; CI, confidence interval; ECOG PS, Eastern cooperative oncology group performance status; IT, immunotherapy; RT, radiotherapy; PFS, progression-free survival.

The median follow-up was 8.7 months (range: 0.1–104.1). The median PFS was 4.5 months (95% CI: 3.9–5), with a 1-year PFS rate of 14.9% (95% CI: 12.1–18.4) (**Figure 3A**).

Subgroup analysis according to primary tumor location showed similar PFS regardless of tumor origin (**Figure 3B**). Patients who were considered unfit for platinum due to comorbidities or accumulated doses of platinum had better prognosis than those with ECOG 2 or platinum refractoriness, with a median PFS of 6.3 and 5.6 months versus, 3.9, and 2.8 months, respectively; *p*-value = 0.004) (**Figure 3C**). Accordingly, ECOG PS was correlated with prognosis and showed a median PFS of 10.1, 5.2, and 3.9 months for ECOG 0 to 2, respectively (*p*-value < 0.001) (**Figure 3D**). Patients who presented with metastatic disease at baseline, treatment compliance, and more than 6 months from the end of radiotherapy also correlated with the prognosis (**Figures 3E–G**).

No differences in PFS were observed regarding previous local treatment based on surgery (4.8 months) or radiotherapy (3.6 months, *p* = 0.147) or the use of taxanes or not in induction treatment (4.7 vs. 4.0 months, *p* = 0.627). Patients who received ICI in any subsequent treatment line had a median PFS of 8.8 months (95% CI: 5.6–11.3) while patients who received subsequent treatments different than ICI had a median PFS of 6 months (95% CI: 2.8–4) (*p*-value = 0.002) (**Figure 3H**).

**Figure 4:**
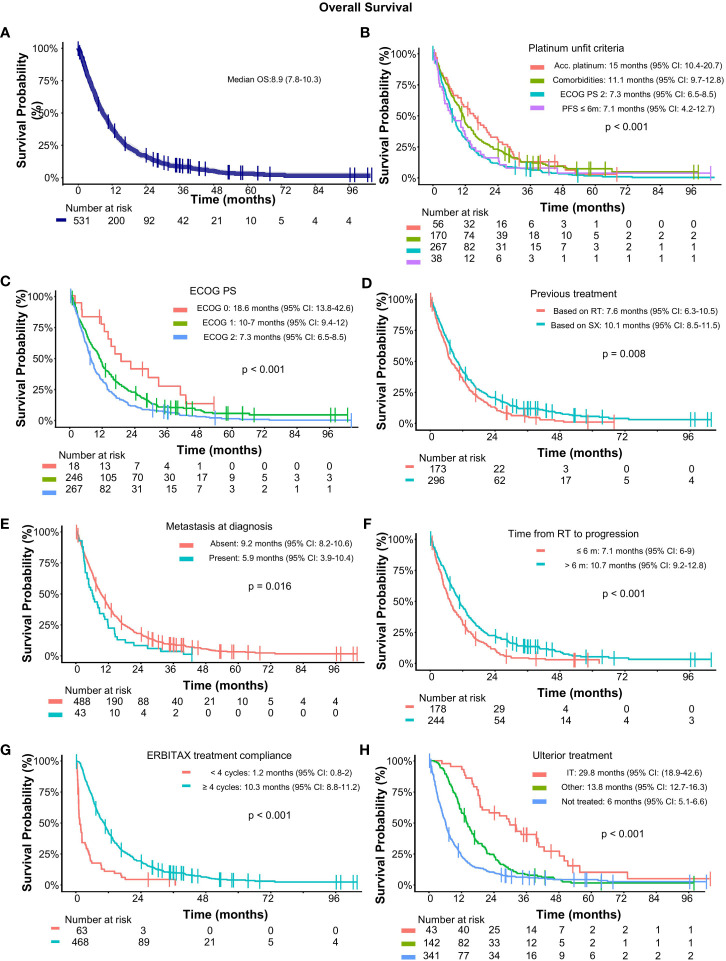
Overall survival in SCCHN treated with cetuximab and paclitaxel as first-line treatment. **(A)** OS for the full dataset; Subgroup analysis of OS according to **(B)** pundit criteria; **(C)** ECOG PS; **(D)** Previoous treatments; **(E)** metastatic status at diagnosis; **(F)** time from radiotherapy; **(G)** ERBITAX treatment compliance; and **(H)** ulterior treatment. Median OS and significance by cox model are displayed within the graphics. Acc, accumulated; CI, confidence interval; ECOG PS, Eastern cooperative oncology group performance status; IT, immunotherapy; RT, radiotherapy.

Overall, 497 (93.6%) patients died. The most common cause of death was inexorable progression of the disease (82.3%). One patient died due to lung infection and another due to sepsis, both secondary to treatment toxicity. The median OS was 8.9 months (95% CI: 7.8–10.3), with a 1-year OS rate of 38.2% (95% CI: 34.2–42.6) (**Figure 4A**). As happened for PFS, patients who were considered unfit for platinum due to comorbidities or accumulated doses of platinum had better OS than those with ECOG 2 or platinum refractoriness (**Figure 4B**).

A subgroup of 11 (2.1%) long-term survivors were disease-free and alive >2 years after the start of study treatment. Only 3 of them underwent subsequent treatments. Multivariate and log-rank analysis revealed that patients with better ECOG performance status, with absence of metastasis at diagnosis, and who are compliant with treatments (**Figures 4C–G**) had better OS. The use of taxanes in induction was not significant for OS.

Patients treated with ICI (nivolumab) in subsequent lines also had better survival with a median OS of 29.8 months (95% CI: 18.9–42.6) and 13.8 months (95% CI: 12.7–16.3) for immunotherapy and other treatments, respectively (*p*-value < 0.001) (**Figure 4H**). After ERBITAX, the median duration of response was 6.9 months (95% CI: 5.3–9.8) for patients who were subsequently treated with ICI and 4.6 months (95% CI: 3.7–6.5) for other treatments (*p* = 0.04).

**Figure 5:**
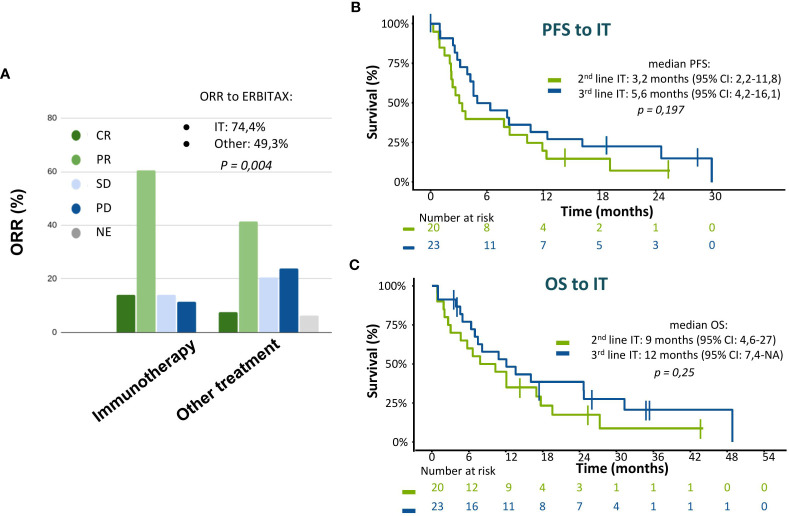
Efficacy of immunotherapy after a first line with ERBITAX regimen. **(A)** ORR to ERBITAX scheme with patients stratified according to ulterior treatment. **(B)** PFS to immunotherapy treatment stratified by treatment line, patients who were treated with immunotherapy as second line vs third line. **(C)** OS to immunotherapy treatment stratified by treatment line, patients who were treated with immunotherapy as second line vs third line. CI, confidence interval; IT, immunotherapy; ORR, objective response rate; OS, overall survival; PFS, progression-free survival.

Patients who received subsequent immunotherapy had significantly better ECOG performance and outcomes to ERBITAX than those who received other ulterior therapies (**Table 2**). Those patients had a good median PFS of 3.2 months (95% CI: 2.2–11.8) when receiving immunotherapy as second line or 5.6 months (95% CI: 4.2–16.1) even in third-line therapy (*p* = 0.197) (**Figure 5**). Patients treated with other drugs instead of ICI mainly received schemes based on carboplatin (43%) or methotrexate (43%). PDL1 determination was performed in few patients (**Table S1**) since the indication for nivolumab was independent of its result. Its approval by the regulatory agencies in our country was in 2017.

**Table 2:** Baseline characteristics for patients who received ulterior immunotherapies or other treatments.

Characteristics; unit		Post Immunotherapy *N* = 43	Post other therapy *N* =142	TTCC-2019-02 ≥1 post therapy *N* = 185	*p*-value
Median age (range); years		66 (35–91)	65 (44–90)	65 (35–91)	0.855
Sex, *n* (%)	Male	32 (74.4)	118 (83.1)	150 (81.1)	0.203
ECOG PS; *n* (%)	0	7 (16.3)	2 (2.1)	10 (5.4)	0.002
	1	17 (39.5)	82 (57.7)	99 (53.5)	
	2	19 (44.2)	57 (40.1)	76 (41.1)	
Stage at diagnosis; *n* (%)	I	3 (7)	7 (4.9)	10 (5.4)	0.784
	II	2 (4.7)	10 (7)	12 (6.5)	
	III	7 (16.3)	28 (19.7)	35 (18.9)	
	IV	29 (67.4)	94 (66.2)	123 (66.5)	
	UK	2 (4.7)	3 (2.1)	5 (2.7)	
Metastasis; *n* (%)		2 (4.7)	13 (9.2)	15 (8.1)	0.526
Smoking habit; *n* (%)	Never smoker	8 (18.6)	13 (9.2)	21 (11.4)	0.301
	Former	20 (46.5)	64 (45.1)	84 (45.4)	
	Current smoker	12 (27.9	54 (38)	66 (35.7)	
	UK	3 (7)	11 (7.7)	14 (7.6)	
PD-L1	Positive	13 (30.2)	5 (3.5)	18 (9.7)	< 0.001
	Negative	7 (16.3)	25 (17.6)	32 (17.3)	
	Not determined	23 (53.5)	112 (78.9)	135 (73)	
Previous surgery; *n* (%)	Yes	32 (74.4)	82 (57.7)	114 (61.6)	0.049
	No	11 (25.6)	60 (42.3)	71 (38.4)	
Cetuximab paclitaxel median treatment duration (range); months		9.1 (0.7–46.4)	5.9 (0.2–27.2)	6.2 (0.2–46.4)	0.002
ORR cetuximab paclitaxel; *n* (%)		32 (74.4)	70 (49.3)	102 (55.1)	0.004
DCR cetuximab paclitaxel; *n* (%)		38 (88.4)	99 (69.7)	137 (74.1)	0.014
Median DoR cetuximab paclitaxel (range); months		6.9 (1.9–19.2)	4.6 (0–35.9)	5.6 (0–35.9)	0.04

Only patients with one or more treatment lines after cetuximab paclitaxel. Those patients who died or had no longer follow-up and did not receive other treatment lines were excluded from the analysis to avoid bias.

DCR, disease control rate; DoR, duration of response; ECOG PS, Eastern Cooperative Oncology Group performance status; ORR, objective response rate.

### 3.5 Safety

The study collected only those AEs that were grade ≥ 3 or led to discontinuation or dose reduction of paclitaxel and/or cetuximab or met criteria for serious AE (SAE). Ninety-four (17.7%) patients experienced grade ≥ 3 treatment-related AEs (toxicities). The most common grade ≥ 3 toxicities were acne-like rash in 36 patients (6.8%) and oral mucositis in 8 patients (1.5%) (**Table 3**). Five (0.9%) patients experienced grade ≥ 3 febrile neutropenia and three (0.6%) had grade ≥ 3 hypomagnesemia, all of whom were properly managed with dose temporary interruptions or reductions. Three patients experienced grade ≥ 3 neurotoxic effects, including paresthesia in two (0.4%) patients and peripheral sensory neuropathy in one (0.2%). Regarding those AEs that led to discontinuation of the regimen, skin rash in 18 patients (3.4%) was the main one.

**Table 3:** Toxicities (treatment-related AEs) to cetuximab plus paclitaxel treatment.

Event; n (%)		Grade ≥ 3	Leading to CP discontinuation	TTCC-2019-02 N = 531
Blood system disorders	Overall	12 (2.3)	4 (0.8)	34 (6.4)
	Neutrophil count decreased	7 (1.3)	1 (0.2)	16 (3)
	Febrile neutropenia	5 (0.9)	2 (0.4)	9 (1.7)
	Anemia	2 (0.4)	2 (0.4)	11 (2.1)
	Leukocytosis	0 (0)	0 (0)	1 (0.2)
Metabolism and nutrition disorders	Overall	3 (0.6)	1 (0.2)	9 (1.7)
	Hypomagnesemia	1 (0.2)	0 (0)	1 (0.2)
	Hypocalcemia	0 (0)	0 (0)	1 (0.2)
	Anorexia	0 (0)	0 (0)	3 (0.6)
General disorders	Overall	12 (2.3)	15 (2.8)	33 (6.2)
	Fatigue	11 (2.1)	9 (1.7)	26 (4.9)
	Infusion-related reactions	1 (0.2)	5 (0.9)	5 (0.9)
	Muscle alterations	0 (0)	0 (0)	1 (0.2)
	Flushing	0 (0)	1 (0.2)	1 (0.2)
Skin and subcutaneous tissue disorders	Overall	52 (9.8)	19 (3.6)	119 (22.4)
	Rash acneiform	47 (8.9)	18 (3.4)	108 (20.3)
	Dry skin	1 (0.2)	0 (0)	5 (0.9)
	Pruritus	0 (0)	0 (0)	1 (0.2)
	Palmo-plantar erythrodysesthesia syndrome	0 (0)	1 (0.2)	3 (0.6)
	Nail toxicity	6 (1.2)	0 (0)	11 (2.1)
	Alopecia	0 (0)	0 (0)	5 (0.9)
	Hypertrichosis	0 (0)	0 (0)	2 (0.4)
Investigations	Overall	4 (0.8)	4 (0.8)	9 (1.7)
	Alkaline phosphatase increased	1 (0.2)	0 (0)	1 (0.2)
	GGT increased	0 (0)	1 (0.2)	1 (0.2)
	Transaminase increased	4 (0.8)	2 (0.4)	7 (1.3)
	Creatinine increased	0 (0)	2 (0.4)	2 (0.4)
Gastrointestinal disorders	Overall	10 (1.9)	9 (1.7)	54 (10.2)
	Oral mucositis	8 (1.5)	8 (1.5)	36 (6.8)
	Esophagitis	1 (0.2)	0 (0)	1 (0.2)
	Emesis	0 (0)	0 (0)	10 (1.9)
	Diarrhea	1 (0.2)	1 (0.2)	13 (2.5)
Infections and infestations	Overall	10 (1.9)	5 (0.9)	18 (3.4)
	Urinary tract infection	1 (0.2)	0 (0)	2 (0.8)
	Respiratory infection	4 (0.8)	2 (0.4)	7 (1.3)
	Sepsis	2 (0.4)	2 (0.4)	2 (0.4)
	Abdominal infection	1 (0.2)	1 (0.2)	1 (0.2)
	Eye infection	2 (0.4)	0 (0)	6 (1.1)
Non-infectious respiratory events	Overall	3 (0.6)	4 (0.8)	6 (1.1)
	Pulmonary toxicity	3 (0.6)	3 (0.6)	5 (0.9)
	Cough	0 (0)	1 (0.2)	1 (0.2)
Tumor-related events	Overall	0 (0)	1 (0.2)	3 (0.6)
	Pain	0 (0)	0 (0)	2 (0.4)
	Fistula	0 (0)	1 (0.2)	1 (0.2)
Others	Overall	5 (0.9)	12 (2.3)	25 (4.7)
	Peripheral sensory neuropathy	5 (0.9)	12 (2.3)	25 (4.7)
	Bone fracture	0 (0)	0 (0)	1 (0.2)

The study collected only those AEs that were grade ≥3, led to discontinuation or dosage reduction of paclitaxel and/or cetuximab, or met criteria for serious AE (SAE).

CP, cetuximab plus paclitaxel; GGT, gamma glutamyl transferase.

## 4 Discussion

The TTCC-2019-02 study showed that ERBITAX regimen is an active first-line treatment option in routine clinical practice for patients with recurrent or metastatic SCCHN who are considered ineligible for platinum-based regimens. The real-world efficacy outcomes agreed with the previous trial that tested ERBITAX (10), in which median PFS and OS were 4.2 and 8.1 months, respectively. Despite the limitations of indirect comparison, the ORR reported in our study was similar to that of first-line platinum/5-FU with cetuximab in the EXTREME trial (37.7% vs. 36%, respectively), in line with the survival outcomes (3). As in a previous report, ERBITAX showed efficacy regardless of previous treatment (10). Treatment compliance was also associated with better outcomes, even though we could not discard bias due to the presence of patients with the worst prognosis among those who had early treatment discontinuation. Twelve percent of patients received less than 4 weeks of ERBITAX, some of whom may be poorly selected to receive chemotherapy. In those who received more than 3 weeks of ERBITAX, a median PFS greater than 5 months was obtained.

The results are especially encouraging considering that a high proportion of patients had an ECOG PS of 2 (50.3%), which is considered a poor prognostic factor associated with inferior OS (13, 14). Having comorbidities or high accumulated doses of platinum as the primary reason for platinum ineligibility showed a significant benefit in survival compared to other platinum unfit criteria, suggesting that the ERBITAX scheme as a first-line treatment should be specially considered for those patient profiles. Similar results have been reported in other retrospective studies (14, 15).

Recently, immunotherapy has changed the paradigm of advanced or metastatic SCCHN. Nivolumab and pembrolizumab in mostly pretreated patients showed an ORR of 13.3% and 14.6% and a median OS of 7.5 and 8.4 months, respectively (5, 6, 16). We observed a remarkably prolonged PFS and OS from the diagnosis of advanced or metastatic disease in patients who received immunotherapy after ERBITAX, even in patients ECOG PS 2, who were not included in the immunotherapy trials.

Real-world studies of immunotherapy after platinum-based chemotherapy reported similar activity to that of phase 3 trials, with a median OS that ranged from 6.5 to 8.7 months (17, 18). Only one previous real-world study with SCCHN patients who received immunotherapy as second-line therapy reported a prolonged median OS of 22.1 months in line with our results (19). DoR and survival were substantially longer in those who received immunotherapy after first-line chemotherapy, suggesting a potential boosting effect of previous chemotherapy (19). Despite their poor prognosis, we achieve that a percentage of patients, although low, can benefit from subsequent lines and improve survival.

The baseline characteristics of the patients who received ulterior immunotherapy suggested a potential selection bias. This may explain the good result of these subsequent lines of treatment. Patients with better outcomes with ERBITAX received immunotherapy in the standard clinical practice. We could not discard the potential conditioning effect of the first-line treatment as already reported in other studies that combine taxanes and cetuximab (4). Retrospective studies propose to explore what is the best combination strategy between schemes with ICI and with cetuximab (20), also given the current positioning of ICI as a first-line treatment for advanced SCCHN.

Regarding tolerability, the rate of permanent discontinuation due to toxicity for cetuximab (8.7%) and paclitaxel (10.2%) was amenable, in line with previous reports (4.6%–13%) (10, 15), and lower than that reported for the EXTREME scheme (20%) (3). The median duration of ERBITAX was shorter than that in the previous clinical trial (6 months) (10) or patients who received the EXTREME scheme (4.5 months) (3, 21), which may indicate that real-world patients discontinue earlier and for reasons other than toxicity. The toxicity profile was similar to that of other clinical trials, with acneiform rash being the most common event (10, 15).

The study is based on the data collected in the clinical reports in the routine practice; thus, the AEs may be under-reported, especially those of low grade. Indirect comparisons of the frequencies of any-grade AEs with previous clinical trials were not feasible as the study only collected AEs leading to treatment discontinuation, those with grade ≥ 3, or those considered serious. The rate of grade ≥ 3 toxicities (17.7%) was much lower than that reported in previous trials (65%) (10). Hypomagnesemia and febrile neutropenia were less frequent than previously reported (10, 15). Two deaths were related to infection processes secondary to the study medication.

The main limitation of this study was its retrospective design, which may have led to an increased rate of missing data, which did not compromise the interpretation of our results. The greatest missing data were reported for smoking habits (5.3%) or stage at diagnosis (1.9%).

Some patients initially with bad performance status due to tumor effect were able to benefit from the response to ERBITAX, improve it, and receive carboplatin-based regimens at next disease progression.

The therapeutic scenario at the time in which the study patients were treated was different from the current one. Some refractory patients or those with contraindications to platinum and positive CPS can now benefit from first-line immunotherapy. Given the good tolerance and effectiveness of ERBITAX, this scheme could be placed in the treatment after progression to chemo-immunotherapy or for those patients who, being refractory to cisplatin, need a fast response.

## 5 Conclusion

Our trial demonstrates that the ERBITAX scheme is feasible as a first-line treatment for advanced SCCHN patients unfit for platinum-based chemotherapy. Treatment with ICI in subsequent lines after ERBITAX implies an important benefit in survival.

## Data availability statement

The raw data supporting the conclusions of this article will be made available by the authors, without undue reservation.

## Ethics statement

The studies involving human participants were reviewed and approved by Ethic Commitee of each participating institution. The patients/participants provided their written informed consent to participate in this study.

## Author contributions

JR-C, BCC and RM were the coordinators of the project and responsible for the final writing of the manuscript. MD was the data coordinator at Spanish Group of Head and Neck Cancer Treatment (TTCC). All authors contributed to study design, patient data acquisition and to the analysis and interpretation of the study results. All authors corrected and approved the final manuscript.
