# Supplementary material for: TTCC-2019-02: real-world evidence of first-line cetuximab plus paclitaxel in recurrent or metastatic squamous cell carcinoma of the head and neck
Source: Front Oncol. 2023 Aug 1;13:1226939. doi: 10.3389/fonc.2023.1226939 (PMC10432957; doi:10.3389/fonc.2023.1226939)
Supplement: Supplementary file 1 [file DataSheet_1.docx]

**Supplementary data**

Table Supplementary 1. ERBITAX patients according to their recruiting site showing geographic distribution of the sample within Spain by CCAA.

| **Hospital** | **Autonomous community** | **n (%)** |
| --- | --- | --- |
| Hospital Universitario Miguel Servet | Aragón | 91 (17.14) |
| Institut Català d'Oncologia L'Hospitalet (Ico) | Cataluña | 79 (14.88) |
| Hospital Universitario Marqués De Valdecilla | Cantabria | 49 (9.23) |
| Institut Català d'Oncologia Badalona (Ico) | Cataluña | 45 (8.47) |
| Hospital Lucus Augusti | Galicia | 36 (6.78) |
| Hospital Universitario 12 De Octubre | Madrid | 30 (5.65) |
| Hospital Clínico San Carlos | Madrid | 29 (5.46) |
| Hospital Universitario De Canarias (H.U.C) | Canarias | 29 (5.46) |
| Complejo Hospitalario Regional De Málaga | Andalucía | 25 (4.71) |
| Hospital Universitari Son Espases | Baleares | 21 (3.95) |
| Centro Oncolóxico De Galicia | Galicia | 21 (3.95) |
| Complejo Hospitalario De Navarra | Navarra | 20 (3.77) |
| Hospital Universitario De Salamanca | Castilla y León | 18 (3.39) |
| Institut Català d'Oncologia Girona (Ico) | Cataluña | 17 (3.20) |
| Hospital Universitario Virgen De Valme | Andalucía | 13 (2.45) |
| Hospital Universitario Virgen De Las Nieves | Andalucía | 8 (1.51) |
